# Sustaining a Research Center on Aging: Strategies for Growth, Resilience, and Impact in Challenging Times

**DOI:** 10.1093/geroni/igaf122.614

**Published:** 2025-12-31

**Authors:** Patricia Heyn

**Affiliations:** Marymount University, Arlington, Virginia, United States

## Abstract

Establishing and sustaining a research center on aging requires strategic vision, institutional support, and adaptability, especially during times of financial uncertainty, shifting institutional and federal priorities, and evolving research landscapes. The Center for Optimal Aging at Marymount University was launched with the goal of fostering interdisciplinary research, enhancing community engagement, and developing mentoring and leadership training models for a diverse aging research workforce. However, launching a new aging center among many funding challenges, administrative turnover, and shifting expectations for research productivity presents unique obstacles. This presentation will outline the critical steps, challenges, and opportunities involved in developing a new research center on aging, including: 1) Strategic planning and institutional alignment – positioning the center’s mission within the broader university and external funding landscape; 2) Building research infrastructure – developing administrative and faculty support for interdisciplinary research while navigating institutional and federal constraints; 3) Sustainability and funding challenges – balancing internal and external funding mechanisms, fostering collaborations, and managing shifting priorities; and 4) Opportunities for growth – leveraging partnerships, faculty mentorship, students and early-career researcher engagement to enhance research capacity. By reflecting on lessons learned in launching and leading a research center in today’s uncertain academic and political environment, this session will provide practical strategies for creating and establishing a new center while positioning the center for long-term impact and success.

